# Liver polyploidy: Dr Jekyll or Mr Hide?

**DOI:** 10.18632/oncotarget.3809

**Published:** 2015-04-13

**Authors:** Géraldine Gentric, Chantal Desdouets

**Affiliations:** Team Cell Cycle and Liver Physiopathology. (1) Inserm, U1016, Institut Cochin, Paris, France. (2) CNRS, UMR 8104, Paris, France. (3) Université Paris Descartes, Sorbonne Paris Cité, Paris, France

Polyploidization is a state in which cells possess more than two sets of homologous chromosomes, which occurs frequently in nature [1]. In Mammals, whole organism polyploidy is usually lethal; however, some tissues develop a certain degree of polyploidy during their normal lifecycle. Polyploid cells are generated because of cell fusion or abnormal cell division (e.g. endoreplication, mitotic slippage, cytokinesis failure). Polyploid cells often appear during late fetal development or following a variety of cellular stressors (eg, mechanical or metabolic stress). Alarmingly, proliferating polyploid cells have been demonstrated to be genetically unstable [1]. Different works have clearly discovered a significant contribution of polyploid intermediates in shaping the composition of cancer genomes. In light of this problem, it is not surprising that mechanisms have evolved to limit proliferation of polyploid contingent: activation of programmed death or senescence pathways as soon as they are generated [1, 2]; elicit immune responses resulting in their elimination [3].

Polyploidy is a common characteristic of the mammalian hepatocytes. Polyploidization occurs mainly during liver development, but also in adults with increasing age or due to cellular stress (eg, surgical resection, toxic exposure) [4]. In the human liver, the majority of polyploid hepatocytes are tetraploid with two nuclei (binucleate cells). Hepatocytes become polyploid usually by failed cytokinesis. During post-natal liver development, the insulin/AKT pathway and the E2F transcription factors have shown to play an important role in the generation of polyploid liver cells [5, 6]. A number of ideas have been proposed to explain the functional significance of physiological polyploidy in the liver. Recent work by Duncan et al., elegantly showed that polyploid hepatocytes can at least promote adaptations to liver injuries by increasing genetic diversity [7]. It is important to note that a long-term consequence of switching to the polyploidization mode during liver pathological growth is still under debate and no study has really defined if polyploidization contributes to liver tumorigenesis. Hepatocellular carcinoma (HCC) is a common and deadly malignancy that is increasing in incidence in developed countries. Non-alcoholic fatty liver disease (NAFLD), the hepatic counterpart of metabolic syndrome, is now recognized as a specific risk factor for HCC development. The spectrum of NAFLD ranges from simple fatty liver to non-alcoholic steatohepatitis (NASH). Of note, NASH cirrhosis is anticipated to be the major etiological factor for HCC in the future as the number of NASH cases continues to increase in parallel with the obesity and diabetes epidemics.

Recently, our group investigated what happened to hepatocyte polyploidization during this pathology setting [8]. In murine models of NAFLD, the parenchyma of fatty livers displayed alterations of the polyploidization process, including the presence of a large proportion of highly polyploid mononuclear cells (≥8n), which are rarely observed in normal hepatic parenchyma. Biopsies from patients with NASH revealed also the presence of this highly polyploid mononuclear contingent; their presence in fatty liver being independent to the severity of fibrosis and preceding HCC development. By taking advantage of primary culture of hepatocytes isolated from NAFLD-mouse models, we demonstrated that the progression of fatty hepatocytes through the S and G2 phases was profoundly altered suggesting that endoreplication is preferentially performed during NAFLD progression. Recent works suggest that pathological polyploidization is an adaptive response to genomic stress. Cells respond to a diverse array of DNA lesions with an evolutionarily conserved DNA damage response. In our system, we assessed whether DNA damage checkpoints were activated. In fatty hepatocytes, we observed that the DNA damage pathway under the control of ATR/p53/p21 signaling triggers the G2/M arrest. It has been well described several decades ago that oxidative stress plays a central role in the progression of NAFLDs. As expected, we found evidence for oxidative stress in NAFLD hepatocytes, both in our *in vitro* and *in vivo* models. This raises the question as to how oxidative stress could be involved in DNA damage promoting pathological polyploidization. To clarify this role, we demonstrated that antioxidant treatments rescue complete cell cycle progression and decrease ATR activation *in vitro*. Finally, does long-term antioxidant treatment modify polyploidization in NAFLD mice liver parenchyma? Remarkably, the proportion of highly polyploidy mononuclear hepatocytes was significantly lower in long term treated NAFLD mice compared to untreated ones, suggesting that impacting on oxidative stress during NAFLD development is sufficient to counteract pathological hepatocyte polyploidization.

**Figure 1:**
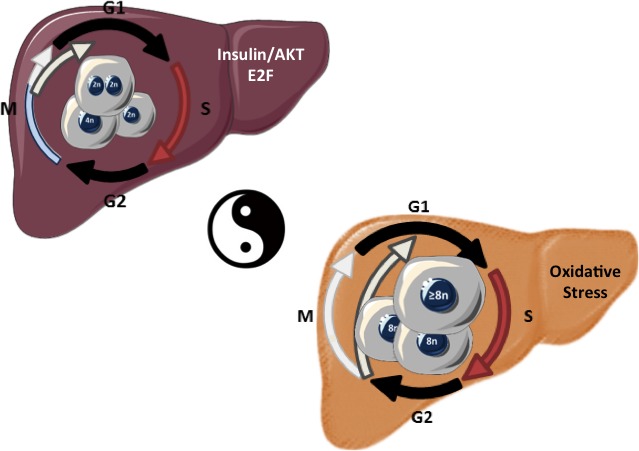
Liver Parenchyma and hepatocyte polyploidy during physiological (left-post-natal) and pathological (right-NAFLD/NASH sequence) growth.

In conclusion, the liver is the only organ that modulates its ploidy content both during its life span and following different types of stress. Collectively, our findings suggest that alteration of ploidy profile can now be considered as a new signature of metabolic liver disorders. Future studies should be aiming to understand the implications of pathological polyploidization during tumorigenesis associated to NAFLD, which is a major public health concern.
